# Draft Genome Sequence of an Erythromycin-Resistant *Propionibacterium acnes* Isolate Recovered from Folliculitis of the Scalp

**DOI:** 10.1128/genomeA.01490-16

**Published:** 2017-01-26

**Authors:** Guillaume Ghislain Aubin, Stanimir Kambarev, Aurélie Guillouzouic, Amir Khammari, Brigitte Dréno, Stéphane Corvec

**Affiliations:** a Bacteriology and Hygiene department, Nantes University Hospital, Nantes, France; b EA3826, Laboratory of Clinical and Experimental Therapeutics of Infections, Institut de Recherche en Santé 2, Nantes, France; c Institut de Recherche en Santé de l'Université de Nantes INSERM U892-CNRS 6299 CRCNA Centre de Recherche en Cancérologie Nantes Angers Université de Nantes, Team 13: Nuclear Oncology Research, Nantes, France; d Institut de Recherche en Santé de l'Université de Nantes INSERM U892-CNRS 6299 CRCNA Centre de Recherche en Cancérologie Nantes Angers Université de Nantes, Team 2: Clinical and Translational Research in Skin Cancer, Nantes, France; e Dermato-Oncology Department, Nantes Hospital, Nantes, France

## Abstract

*Propionibacterium acnes* is now well-known and recognized for its implication in the pathogenesis of acne vulgaris. Here, we report the draft genome sequence of an erythromycin-resistant *P. acnes* strain isolated from a case of folliculitis of the scalp belonging to phylotype IA1 and sequence type 18 (ST18).

## GENOME ANNOUNCEMENT

*Propionibacterium acnes* is an aerotolerant anaerobic Gram-positive bacterium constituting a significant part of the human skin microbiota (1). Although *P. acnes* is considered to be commensal and is likely to be found on all healthy adult skin (2), this bacterium is clearly well-known for its implication in the pathogenesis of acne or folliculitis (3–6). Moreover, *P. acnes* is clearly underestimated in medical device-related infections and sometimes considered a contaminant (7, 8). Using different molecular typing methods (multilocus sequence typing [MLST]/sequence-based strain typing [SLST]), *P. acnes* strains have been subdivided into six main phylogenetic types: IA1, IA2, IB, IC, II, and III (9, 10). In the acne field, phylotype/SLST type IA1/A1 is the most frequent lineage found (9, 11). In the context of this skin disorder, erythromycin, clindamycin, and tetracycline resistances have been extensively reported over the years (12, 13).

Here, we present the draft genome sequence of *Propionibacterium acnes* HB strain isolated from a patient suffering from folliculitis at Nantes University Hospital, France.

*P. acnes* HB was grown overnight at 37°C on a Schaedler agar plate (Oxoid, United Kingdom) under an anaerobic atmosphere. Genomic DNA was extracted using a DNeasy blood and tissue kit (Qiagen Gmbh, Germany), as described previously (14). A paired-end library was prepared with NEBNext Ultra DNA library prep kit for Illumina (NEB) and sequenced (2 × 150 bp) on a MiSeq sequencer (Illumina, USA). *De novo* assembly was performed with Velvet 1.2.10 and VelvetOptimiser 2.2.5 (optimal hash value = 127). A total of 4,394,492 reads were assembled into 21 contigs with an average coverage of 205×. Contig reordering and annotation were performed with Mauve 2.3.1 and the NCBI Prokaryotic Genome Automatic Annotation Pipeline (PGAAP), respectively (15, 16). Sequence alignment and comparison were performed with CLC Sequence Viewer 7.0 and BLAST. Average nucleotide identity (ANI) to the *P. acnes* reference strain KPA171202 was calculated using Oat 0.91 (17).

The draft genome of strain HB (GenBank accession no. MDGS00000000) contains 2,328 genes, 2,275 coding sequences (CDSs), 45 tRNAs, four rRNAs, and four noncoding RNAs. The G+C content was 60.0%, and the ANI value was 99.1%.

Although *Propionibacterium* species on healthy human skin could be highly diverse (18, 19), an increased frequency of IA1/A1 phylotype/SLST has been observed, especially in case of severe acne (5). Despite a conserved core genome of 83% for *P. acnes* (20), with more than 100 genome sequences of *P. acnes* from different clonal complexes or sequence types at that time, genome analysis compared with clinical data can help to better understand how the inflammatory potential of *P. acnes* sequenced strains may influence the severity of skin lesions (4).

*P. acnes* HB belongs to ST18, phylotype IA1, and SLST A1. Sequence analysis revealed that the 23S rRNA gene is mutated at position 2059 (according to the *Escherichia coli* numbering), thus leading to a high-level resistance to erythromycin. This draft genome will be also used for studying the impact of different virulence factors, especially lipase or hyaluronate lyase on skin innate immunity (21).

### Accession number(s).

This whole-genome shotgun project has been deposited at DDBJ/EMBL/GenBank under the accession number MDGS00000000.

## References

[1] Leccia MT, Auffret N, Poli F, Claudel JP, Corvec S, Dreno B 2015 Topical acne treatments in Europe and the issue of antimicrobial resistance. J Eur Acad Dermatol Venereol 29:1485–1492. doi:10.1111/jdv.12989. 25677763

[2] Jahns AC, Eilers H, Alexeyev OA 2016 Transcriptomic analysis of *Propionibacterium acnes* biofilms *in vitro*. Anaerobe 42:111–118. doi:10.1016/j.anaerobe.2016.10.001. 27725231

[3] Lomholt HB, Kilian M 2010 Population genetic analysis of *Propionibacterium acnes* identifies a subpopulation and epidemic clones associated with acne. PLoS One 5:e12277. doi:10.1371/journal.pone.0012277. 20808860 PMC2924382

[4] Jasson F, Nagy I, Knol AC, Zuliani T, Khammari A, Dréno B 2013 Different strains of *Propionibacterium acnes* modulate differently the cutaneous innate immunity. Exp Dermatol 22:587–592. doi:10.1111/exd.12206. 23947673

[5] Saint-Jean M, Frenard C, Le Bras M, Aubin GG, Corvec S, Dréno B 2015 Testosterone-induced acne fulminans in twins with Kallmann’s syndrome. JAAD Case Rep 1:27–29. doi:10.1016/j.jdcr.2014.10.005. 27075133 PMC4802552

[6] Hersle K, Mobacken H, Möller A 1979 Chronic non-scarring folliculitis of the scalp. Acta Derm Venereol 59:249–253. 87084

[7] Bémer P, Corvec S, Tariel S, Asseray N, Boutoille D, Langlois C, Tequi B, Drugeon H, Passuti N, Touchais S 2008 Significance of *Propionibacterium acnes*-positive samples in spinal instrumentation. Spine 33:E971–E976. doi:10.1097/BRS.0b013e31818e28dc. 19092607

[8] Portillo ME, Corvec S, Borens O, Trampuz A 2013 *Propionibacterium acnes*: an underestimated pathogen in implant-associated infections. BioMed Res Int 2013:804391. doi:10.1155/2013/804391. 24308006 PMC3838805

[9] Aubin GG, Portillo ME, Trampuz A, Corvec S 2014 *Propionibacterium acnes*, an emerging pathogen: from acne to implant-infections, from phylotype to resistance. Med Mal Infect 44:241–250. doi:10.1016/j.medmal.2014.02.004. 24656842

[10] Scholz CFP, Jensen A, Lomholt HB, Brüggemann H, Kilian M 2014 A novel high-resolution single locus sequence typing scheme for mixed populations of *Propionibacterium acnes in vivo*. PLoS One 9:e104199. doi:10.1371/journal.pone.0104199. 25111794 PMC4128656

[11] Sadhasivam S, Sinha M, Saini S, Kaur SP, Gupta T, Sengupta S, Ghosh S, Sardana K 2016 Heterogeneity and antibiotic resistance in *Propionibacterium acnes* isolates and its therapeutic implications: blurring the lines between commensal and pathogenic phylotypes. Dermatol Ther 29:451–454. doi:10.1111/dth.12391. 27424878

[12] Oprica C, Löfmark S, Lund B, Edlund C, Emtestam L, Nord CE 2005 Genetic basis of resistance in *Propionibacterium acnes* strains isolated from diverse types of infection in different European countries. Anaerobe 11:137–143. doi:10.1016/j.anaerobe.2005.01.005. 16701544

[13] Dreno B, Thiboutot D, Gollnick H, Bettoli V, Kang S, Leyden JJ, Shalita A, Torres V, Global Alliance to Improve Outcomes in Acne 2014 Antibiotic stewardship in dermatology: limiting antibiotic use in acne. Eur J Dermatol 24:330–334. doi:10.1684/ejd.2014.2309. 24721547

[14] Aubin GG, Kambarev S, Bémer P, Lawson PA, Corvec S 2016 Draft genome sequence of highly rifampin-resistant *Propionibacterium namnetense* NTS 31307302^T^ isolated from a patient with a bone infection. Genome Announc 4(4):e00819-16. doi:10.1128/genomeA.00819-16. 27516511 PMC4982290

[15] Darling ACE, Mau B, Blattner FR, Perna NT 2004 Mauve: multiple alignment of conserved genomic sequence with rearrangements. Genome Res 14:1394–1403. doi:10.1101/gr.2289704. 15231754 PMC442156

[16] Angiuoli SV, Gussman A, Klimke W, Cochrane G, Field D, Garrity G, Kodira CD, Kyrpides N, Madupu R, Markowitz V, Tatusova T, Thomson N, White O 2008 Toward an online repository of Standard Operating Procedures (SOPs) for (meta)genomic annotation. OMICS 12:137–141. doi:10.1089/omi.2008.0017. 18416670 PMC3196215

[17] Lee I, Kim YO, Park SC, Chun J 2015 OrthoANI: an improved algorithm and software for calculating average nucleotide identity. Int J Syst Evol Microbiol. doi:10.1099/ijsem.0.000760. 26585518

[18] Aubin GG, Bémer P, Kambarev S, Patel NB, Lemenand O, Caillon J, Lawson PA, Corvec S 2016 *Propionibacterium namnetense* sp. nov., isolated from a human bone infection. Int J Syst Evol Microbiol. doi:10.1099/ijsem.0.001204. 27259292

[19] Lomholt HB, Kilian M 2014 Clonality and anatomic distribution on the skin of antibiotic resistant and sensitive *Propionibacterium acnes*. Acta Derm Venereol 94:534–538. doi:10.2340/00015555-1794. 24577497

[20] Scholz CFP, Brüggemann H, Lomholt HB, Tettelin H, Kilian M 2016 Genome stability of *Propionibacterium acnes*: a comprehensive study of indels and homopolymeric tracts. Sci Rep 6:20662. doi:10.1038/srep20662. 26857276 PMC4746626

[21] Brüggemann H, Lomholt HB, Kilian M 2012 The flexible gene pool of *Propionibacterium acnes*. Mob Genet Elements 2:145–148. doi:10.4161/mge.21204. 23061021 PMC3463471

